# Quality of observational studies in prestigious journals of occupational medicine and health based on Strengthening the Reporting of Observational Studies in Epidemiology (STROBE) Statement: a cross-sectional study

**DOI:** 10.1186/s13104-018-3367-9

**Published:** 2018-05-02

**Authors:** Javad Aghazadeh-Attari, Kazhal Mobaraki, Jamal Ahmadzadeh, Behnam Mansorian, Iraj Mohebbi

**Affiliations:** 1 Department of neurosurgery, Social Determinants of Health Research Center, Urmia University of Medical Sciences, Urmia, Iran; 2 Social Determinants of Health Research Center, Urmia University of Medical Sciences, Urmia, Iran; 3 Social Determinants of Health Research Center, Occupational Medicine Center, Urmia University of Medical Sciences, Resalat Street, Urmia, Iran

**Keywords:** STROBE statement, Observational studies, Occupational medicine and health, Authors, Reviewers, Editors, Journals

## Abstract

**Objective:**

The present study applied the Strengthening the Reporting of Observational Studies in Epidemiology (STROBE) statement to observational studies published in prestigious occupational medicine and health journals.

**Results:**

A total of 60 articles was evaluated. All sub-items were reported in 63.74% (95% confidence interval [CI], 56.24–71.24%), not reported in 29.70% (95% CI, 20.2–39.2%), and not applicable in 6.56% (95% CI, 4.86–8.26%) of the studies. Of the 45 sub-items investigated in this survey, eight were reported 100% of the time, 13 were addressed in more than 90% of the articles, 22 were included in more than 75% of the studies, and 27 sub-items were applied in more than 50% of the articles published in the journals included in this study.

**Electronic supplementary material:**

The online version of this article (10.1186/s13104-018-3367-9) contains supplementary material, which is available to authorized users.

## Introduction

Observational studies have an important role in researching the benefits and harms of medical interventions [1]. The results of these studies should be reported as transparently as possible “*so that readers can follow what was planned, what was done, what was found, and what conclusions were drawn*” [2]. The credibility of a research depends on a critical assessment by others, in the study design, conduct, and analysis of it [3]. In order to assess the strengths and weaknesses of the evidence from the observational studies von Elm et al. designed in 2007 a 22-item checklist to assist with clear reporting of observational studies called: the Strengthening the Reporting of Observational Studies in Epidemiology (STROBE) Statement [2]. This checklist includes a description of methodological items and instructions on how to use them to transparently report observational studies. Several extensions of these statements have been published with additional recommendations for specialized fields of research—for example, the Strengthening the Reporting of Observational Studies in Epidemiology for Newborn Infection (STROBE-NI) [4] and Strengthening the Reporting of Observational Studies in Epidemiology—nutritional epidemiology (STROBE-nut) [5].

To our knowledge, the quality of reporting in occupational observational studies has not been assessed. Observational study designs are often used in studies published in occupational medicine and health journals. Therefore, the present study investigated the quality of occupational observational studies reporting post-STROBE statements. Application of these recommendations by upcoming observational studies in occupational health and medical journals, will increase the value of new data and avoid wasted research.

## Main text

### Methods

#### Journal identification

We conducted a cross-sectional study and selected four top occupational medicine and health journals with high-impact factors (IFs) based on the Information Sciences Institute (ISI) in the Web of Knowledge (http://www.webofknowledge.com, date of access 14 Aug 2017) as of 2016, including journals in the first quartile in category (Q1) of scientific journal ranking (http://www.scimagojr.com, date of access 14 Aug 2017) among the most prestigious and impactful occupational medicine and health journals indexed in international databases. In addition, we checked the `*Instruction for authors*’ section of the websites for each of the included journals to determine if they contained the STROBE statement for authors or endorsed it as a guideline for reporting observational research articles (Table 1).

**Table 1:** Top four occupational medicine and health journals and impact factor and STROBE endorsement

Scientific journal ranking	Full journal title (NLM title abbreviation)	Impact factor in 2016	Endorse STROBE?
(Q_1_)	Scandinavian Journal of Work, Environment and Health (Scand J Work Environ Health)	4.071	Yes
(Q_1_)	Occupational and Environmental Medicine (Occup Environ Med)	3.912	Yes
(Q_1_)	Journal of Occupational and Environmental Medicine (J Occup Environ Med)	1.861	No
(Q_1_)	American Journal of Industrial Medicine (Am J Ind Med)	1.732	No

#### Study search and selection

We first evaluated the archives from January 1 until July 19, 2017, to retrieve observational studies (cohort, case–control, and cross-sectional studies) published in each of the selected journals. During this period, we identified enough cohort and cross sectional studies, but did not find enough case–control studies; thus, only for this type of study, we expanded the search range from January 1, 2016, to July 19, 2017. The search revealed 188 observational articles.

Second, the types of articles were classified according to the journal in which they were published. Next, a specific number was assigned to each selected article and, based on random numbers table, we randomly selected 15 observational (five each cohort, case–control, and cross sectional) studies published in each of the four prestigious occupational medicine and health journals. Accordingly, we enrolled 60 observational articles in our study, which were randomly assigned to two reviewers without blinding the name of the journal or the authors of the articles. The reviewers independently made decisions regarding the number of items from the STROBE check-list which were addressed in the selected studies.

#### Reliability and validity adherence

To check the judgment of two reviewers regarding the quality of observational studies, we conducted a pilot study as follows: first, among the articles that were previously retrieved, an article was selected randomly and the strengths and weaknesses of its evidence were assessed based on the predetermined checklist of items. Any disagreement over the items was discussed to reach the same interpretation of the checklist in order to increase the reliability between the two reviewers.

Second, two articles were selected randomly from among all the selected studies for the two reviewers to evaluate the checklist items based on three choices for each sub-item (reported/not reported/not applicable). This work was done in order to calculate an indicator of the reliability between two reviewers, the KAPPA statistic, in evaluating the items of the STROBE checklist in the selected articles. The KAPPA statistic was calculated according to the formula below:

$${\text{KAPPA}} = \frac{{({\text{Percent}}\;{\text{agreement}}\;{\text{observed}}) - ({\text{Percent}}\;{\text{agreement expected}}\;{\text{by}}\;{\text{chance}}\;{\text{alone}}\;)}}{{100\% - ({\text{Percent}}\;{\text{agreement}}\;{\text{expected}}\;{\text{by}}\;{\text{chance}}\;{\text{alone}})}} =$$

Landis and Koch suggested that a Kappa greater than 0.75 represents excellent agreement beyond chance, a Kappa below 0.40 represents poor agreement and a Kappa of 0.40–0.75 represents intermediate to good agreement [6]. Based on Additional file 1: Table S1 and Additional file 2: Table S2, the Kappa statistic between reviewers A and B in this study was 53%

$${\textit{KAPPA}} = \frac{{76.7 - 50.86\% }}{{100 - 50.86\% }} = 0.53.$$

In order to assess the validity of the reviewers’ judgments in this cross-sectional study, reviewers A (J. Ah) and B (K. Mo) read and scored the included observational articles and independently made decisions regarding the quality of reporting in each observational study. Any disagreements were resolved by adjudication with the third (I. Mo) and fourth authors (B. Ma).

#### Data analysis

Statistical analyses were conducted using IBM SPSS Statistics for Windows, version 20.0 (IBM Corp; Armonk, NY, USA). The absolute frequencies and percentages of each sub-item in the selected articles were addressed. The total percentage for all sub-items (1–22) was reported (Table 2).

**Table 2:** Percentage of items in the STROBE checklist which were addressed in cohort, case control, and cross-sectional studies published in four top scientific occupational journals in 2017

Item	Recommendation	Reported n (%)	Not reported n (%)	Not applicable n (%)
Title and abstract				
1a	Indicate the study’s design with a commonly used term in the title or the abstract	39 (65.0)	21 (35.0)	0 (0.0)
1b	Provide in the abstract an informative and balanced summary of what was done and what was found	45 (75.0)	15 (25.0)	0 (0.0)
Introduction				
2	Explain the scientific background and rationale for the investigation being reported	60 (100.0)	0 (0.0)	0 (0.0)
3	State specific objectives, including any pre-specified hypotheses	53 (88.3)	7 (11.7)	0 (0.0)
Methods				
4	Present key elements of study design early in the paper	31 (51.7)	29 (48.3)	0 (0.0)
5	Describe the setting, locations, and relevant dates, including periods of recruitment, exposure, follow-up, and data collection	54 (90.0)	6 (10.0)	0 (0.0)
6a	Give the eligibility criteria	51 (85.0)	7 (11.7)	2 (3.3)
6b	Describe methods of follow-up	56 (93.3)	4 (6.7)	0 (0.0)
6c	Give matching criteria	7 (11.7)	3 (5)	50 (83.3)
6d	Give number of exposed and unexposed in matched studies	4 (6.7)	4 (6.7)	52 (86.7)
7a	Clearly, define all outcomes	60 (100.0)	0 (0.0)	0 (0.0)
7b	Clearly, define all exposures	53 (88.3)	6 (10.0)	1 (1.7)
7c	Clearly, define all predictors	55 (91.7)	3 (5.0)	2 (3.3)
7d	Clearly, define all potential confounders	41 (68.3)	19 (31.7)	0 (0.0)
7e	Clearly, define all effect modifiers	25 (41.7)	35 (58.3)	0 (0.0)
8a	give sources of data	60 (100.0)	0 (0.0)	0 (0.0)
8b	details of methods of assessment (measurement)	52 (86.7)	8 (13.3)	0 (0.0)
9	Describe any efforts to address potential sources of bias	20 (33.3)	40 (66.7)	0 (0.0)
10	Explain how the study size was arrived at	21 (35.0)	22 (36.7)	17 (28.3)
11	If applicable, describe which groupings were chosen and why	46 (76.7)	9 (15.0)	5 (8.3)
12a	Describe all statistical methods, including those used to control for confounding	60 (100)	0 (0.0)	0 (0.0)
12b	Describe all statistical software	38 (63.3)	22 (36.7)	0 (0.0)
12c	Describe any methods used to examine subgroups and interactions	13 (21.7)	45 (75.0)	2 (3.3)
12d	Explain how missing data were addressed	29 (48.3)	29 (48.3)	2 (3.3)
12e	If applicable, explain how loss to follow-up was addressed	23 (38.3)	24 (40)	13 (21.7)
12f	Describe any sensitivity analyses	5 (8.3)	55 (91.7)	0 (0.0)
Results				
13a	Report numbers of individuals at each stage of study	28 (46.7)	26 (43.3)	6 (10.0)
13b	Give reasons for non-participation at each stage	4 (6.7)	56 (93.3)	0 (0.0)
13c	Consider use of a flow diagram	28 (46.7)	32 (53.3)	0 (0.0)
14a	Give characteristics of study participants	44 (73.3)	16 (26.7)	0 (0.0)
14b	Indicate number of participants with missing data for each variable of interest	8 (13.3)	48 (80.0)	4 (6.7)
14c	Summarise follow-up time (e.g., average and total amount)	21 (35.0)	38 (63.3)	1 (1.7)
15	Report numbers of outcome events or summary measures	60 (100.0)	0 (0.0)	0 (0.0)
16a	Give unadjusted estimates	36 (60.0)	24 (40.0)	0 (0.0)
16b	Give confounder-adjusted estimates	26 (43.3)	34 (56.7)	0 (0.0)
16c	Give estimates precision/confidence interval	54 (90.0)	6 (10.0)	0 (0.0)
16d	Report category boundaries when continuous variables were categorized	48 (80.0)	4 (6.7)	8 (13.3)
16e	If relevant, consider translating estimates of relative risk into absolute risk for a meaningful time period	14 (23.3)	34 (56.7)	12 (20.0)
17	Report other analyses done—e.g. analyses of subgroups and interactions and sensitivity analyses	14 (23.3)	46 (76)	0 (0.0)
Discussion				
18	Summarise key results with reference to study objectives	60 (100.0)	0 (0.0)	0 (0.0)
19	Discuss limitations of the study, taking into account sources of potential bias or imprecision. Discuss both direction and magnitude of any potential bias	53 (88.3)	7 (11.7)	0 (0.0)
20a	Give a cautious interpretation of results considering objectives	60 (100.0)	0 (0.0)	0 (0.0)
20b	Explain results from similar studies	60 (100.0)	0 (0.0)	0 (0.0)
21	Discuss the generalisability (external validity) of the study results	46 (76.7)	14 (23.3)	0 (0.0)
Other information				
22	Give the source of funding and the role of the funders for the present study	56 (93.3)	4 (6.7)	0 (0.0)
1–22	Total	63.74	29.70	6.56

The STROBE statement is the guidelines for reporting observational studies, defined as Strengthening the Reporting of Observational Studies in Epidemiology

### Results

**Fig. 1:**
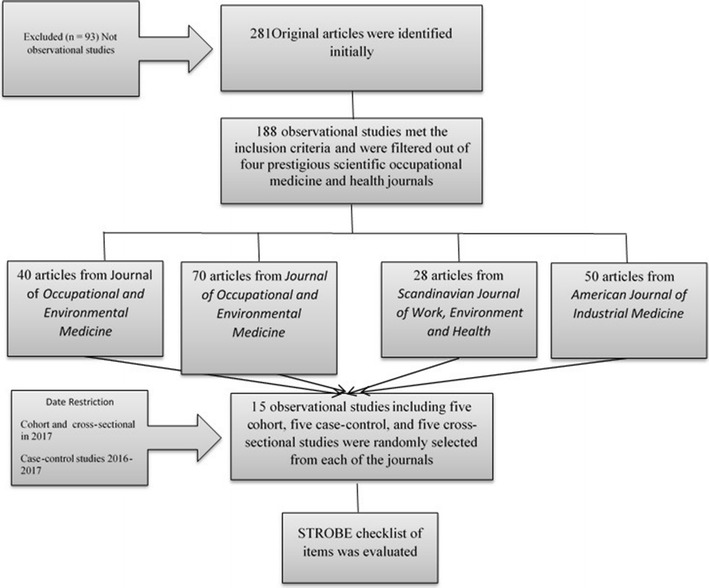
The overview of study design. STROBE checklist of items was addressed in prestigious scientific occupational medicine and health journals, 2016–2017

Figure 1 shows the flow diagram of the search process and the numbers of observational articles included at each stage of the study published in prestigious scientific occupational medicine and health journals in 2016–2017. During the search process, of 281 original articles, 93 were not observational studies and were excluded from the study. Finally, we selected 60 observational studies from four prestigious scientific occupational medicine and health journals, including Scand J Work Environ Health, Occup Environ Med, J Occup Environ Med, and Am J Ind Med.

The absolute frequencies and percentages of items and sub-items addressed by these studies are summarized in Table 2. The sub-items were reported in 63.74% (95% confidence interval[CI], 56.24–71.24%), not reported in 29.70% (95% CI, 20.2–39.2%), and not applicable in 6.56% (95% CI, 4.86–8.26%) of the cohort, case–control, and cross sectional studies. This table also shows that, of the 45 sub-items investigated in this survey, collectively, eight sub-items were reported 100% of the time, 13 sub-items were addressed in more than 90% of the articles, 22 sub-items were included in more than 75% of the studies, and 27 sub-items were applied in more than 50% of the articles assessed from the journals included in this study.

Additional file 3: Figure S3 shows the percentages of adequately reported STROBE sub-items in articles published in the top four occupational medicine and health journals. In this figure, sub-items including 2-Introduction, 7a-Outcomes, 8a-Sources of data, 12a-Control for confounding, 15-N of outcome events, 18-Key results, 20a-Interpretation, and 20b-Similar studies were all reported in the articles published in the four prestigious journals included in this study.

Additional file 4: Figure S4 shows the percentages of STROBE sub-items not reported in articles published in the top four occupational medicine and health journals. Collectively, the five sub-items with the lowest reporting in the articles were 12f-Sensitivity analyses, 13b-Non-participation, 17-Other analyses, 14b-N of participants with missing data, and 12c-Interactions.

Additional file 5: Figure S5 shows that among 45 sub-items in the STROBE statement, those such as 12e-Loss to follow-up, 6d-N of exposed and unexposed, and 6c-matching criteria were the most common not applicable items in the articles included in the present study.

### Discussion

The STROBE statement provides valuable recommendations for all authors when reporting the results of analytical observational studies, supports editors and reviewers when considering these articles for publication, and helped readers to critically appraise published articles [2]. In total, approximately 63.47% of the items and sub-items in the STROBE checklist were reported in cohort, case–control, and cross-sectional studies published in the four journals of occupational medicine and health included in the present study, 11 years after the dissemination of the STROBE statement. However, numerous observational studies are published in less fastidious peer-reviewed occupational medicine and health journals. Thus, it is expected that the quality of reporting of such studies is poorer than that reported in the present survey, although the results of the present study indicate that even this quality is not sufficient. To our knowledge, this study is the first to assess the quality of reporting of observational studies in occupational medicine and health literature; however, similar work has been performed in other disciplines; for example, Hendriksma et al. [7]. in 2016 con-ducted a similar study to evaluate the quality of reporting of observational studies in otorhinolaryngology based on the STROBE statement. They reported that the articles in the top five general medical journals reported a mean of 69.2% (95% CI 65.8 ± 72.7%) of items, compared to 51.4% (95% CI 47.7 ± 55.0%) in the top five ear, nose, and throat journals. These results are similar to the mean of 63.74% (95% CI 56.24–71.24%) obtained in our study. Jeelani et al. [8]. conducted a similar study in 2014 to assess the quality of reporting of cross-sectional studies published in the *Indian Journal of Community Medicine* by evaluating the extent to which they adhered to the STROBE statement. In this study, the most frequently reported checklist items included the summary of what was done and what was found in the abstract, background/rationale, objectives, setting, outcome data, key results in the discussion, and interpretation of results. In our study (Additional file 2: Table S2) the most frequently reported STROBE checklist items were the explanation of the scientific background and rationale for the investigation being reported; clearly defining all outcomes; providing sources of data; and describing all statistical methods, including those used to control for confounding; reporting the numbers of outcome events or summary measures; summarizing key results with reference to study objectives; providing a cautious interpretation of results considering the objectives, and explaining the results of similar studies. The findings in our study are consistent with those reported in the previous study [8, 9].

In 2016, Agha et al. [9] assessed the compliance of observational studies in plastic surgery using the STROBE statement checklist. The average STROBE score in his study was 12.4 (range 2–20.1) with a standard deviation of 3.36. This mean in plastic surgery articles is not satisfactory and lower than the average obtained from the occupational medicine and health journals evaluated in the present study.

The results of this study reveal that the quality reporting of observational studies published in the most prestigious occupational medicine and health journals is yet not clear and desirable enough. Thus, this issue should be the focus of the both authors’ and editors’ special attention when reporting and/or reviewing the reports of observational studies.

## Limitations

- First, the random selection of cohort, case–control, and cross sectional studies from prestigious occupational medicine and health journals may result in a selection bias in the results.
- Second, the limited number of articles selected and evaluated in this survey, may in-crease the possibility of random error.
- Third, the quality and accuracy of the STROBE sub-items addressed in the selected articles depended primarily on the judgment of the reviewer, which increased the probability of information bias in the results.

## Additional files

**Additional file 1: Table S1.** Percent agreement between reviewers A and B.

**Additional file 2: Table S2.** Percent agreement between reviewers A and B expected by chance alone.

**Additional file 3: Figure S1.** Percentages of adequately reported STROBE sub-items in articles published in the top four occupational medicine and health journals.

**Additional file 4: Figure S2.** Percentages of STROBE items not adequately reported in articles published in the top four occupational medicine and health journals.

**Additional file 5: Figure S5.** Percentages of not applicable STROBE items in articles published in the top four occupational medicine and health journals.

## Abbreviations

STROBE: The Strengthening the Reporting of Observational Studies in Epidemiology
IFs: impact factors
ISI: The Information Sciences Institute
Q1: in the first quartile in category
Scand J Work Environ Health: Scandinavian Journal of Work, Environment and Health
Occup Environ Med: Occupational and Environmental Medicine
J Occup Environ Med: Journal of Occupational and Environmental Medicine
Am J Ind Med: American Journal of Industrial Medicine
