# Supplementary material for: Quality of observational studies in prestigious journals of occupational medicine and health based on Strengthening the Reporting of Observational Studies in Epidemiology (STROBE) Statement: a cross-sectional study
Source: BMC Res Notes. 2018 May 2;11:266. doi: 10.1186/s13104-018-3367-9 (PMC5932818; doi:10.1186/s13104-018-3367-9)
Supplement: Supplementary file 1 — Additional file 1: Table S1. Percent agreement between reviewers A and B. [file 13104_2018_3367_MOESM1_ESM.docx]

|  | | Reviewers A | | |  |
| --- | --- | --- | --- | --- | --- |
|  |  | Reported | Not reported | Not applicable | Totals sub-items by B |
| Reviewers B | Reported | 51 | 4 | 1 | 56 |
|  | Not reported | 9 | 15 | 0 | 24 |
|  | Not applicable | 3 | 4 | 3 | 10 |
|  | Totals sub-items by A | 63 | 23 | 4 | 90 |
| Percent agreement observed:$\frac{51+15+3}{90}\times100=76.7\%$ | | | | | |
