# Supplementary material for: Quality of observational studies in prestigious journals of occupational medicine and health based on Strengthening the Reporting of Observational Studies in Epidemiology (STROBE) Statement: a cross-sectional study
Source: BMC Res Notes. 2018 May 2;11:266. doi: 10.1186/s13104-018-3367-9 (PMC5932818; doi:10.1186/s13104-018-3367-9)
Supplement: Supplementary file 2 — Additional file 2: Table S2. Percent agreement between reviewers A and B expected by chance alone. [file 13104_2018_3367_MOESM2_ESM.docx]

|  | | Reviewers A | | |  |
| --- | --- | --- | --- | --- | --- |
|  |  | Reported | Not reported | Not applicable | Totals sub-items by B |
| Reviewers B | Reported | 39.200 | 14.311 | 2.488 | 56 |
|  | Not reported | 16.800 | 6.133 | 1.066 | 24 |
|  | Not applicable | 7.000 | 2.555 | 0.444 | 10 |
|  | Totals sub-items by A | 63 | 23 | 4 | 90 |
| Percent agreement expected by chance alone:$\frac{39.200+6.133+0.444}{90}\times100=50.86\%$ | | | | | |
