# Supplementary figures and images for: Quality of observational studies in prestigious journals of occupational medicine and health based on Strengthening the Reporting of Observational Studies in Epidemiology (STROBE) Statement: a cross-sectional study
Source: BMC Res Notes. 2018 May 2;11:266. doi: 10.1186/s13104-018-3367-9 (PMC5932818; doi:10.1186/s13104-018-3367-9)

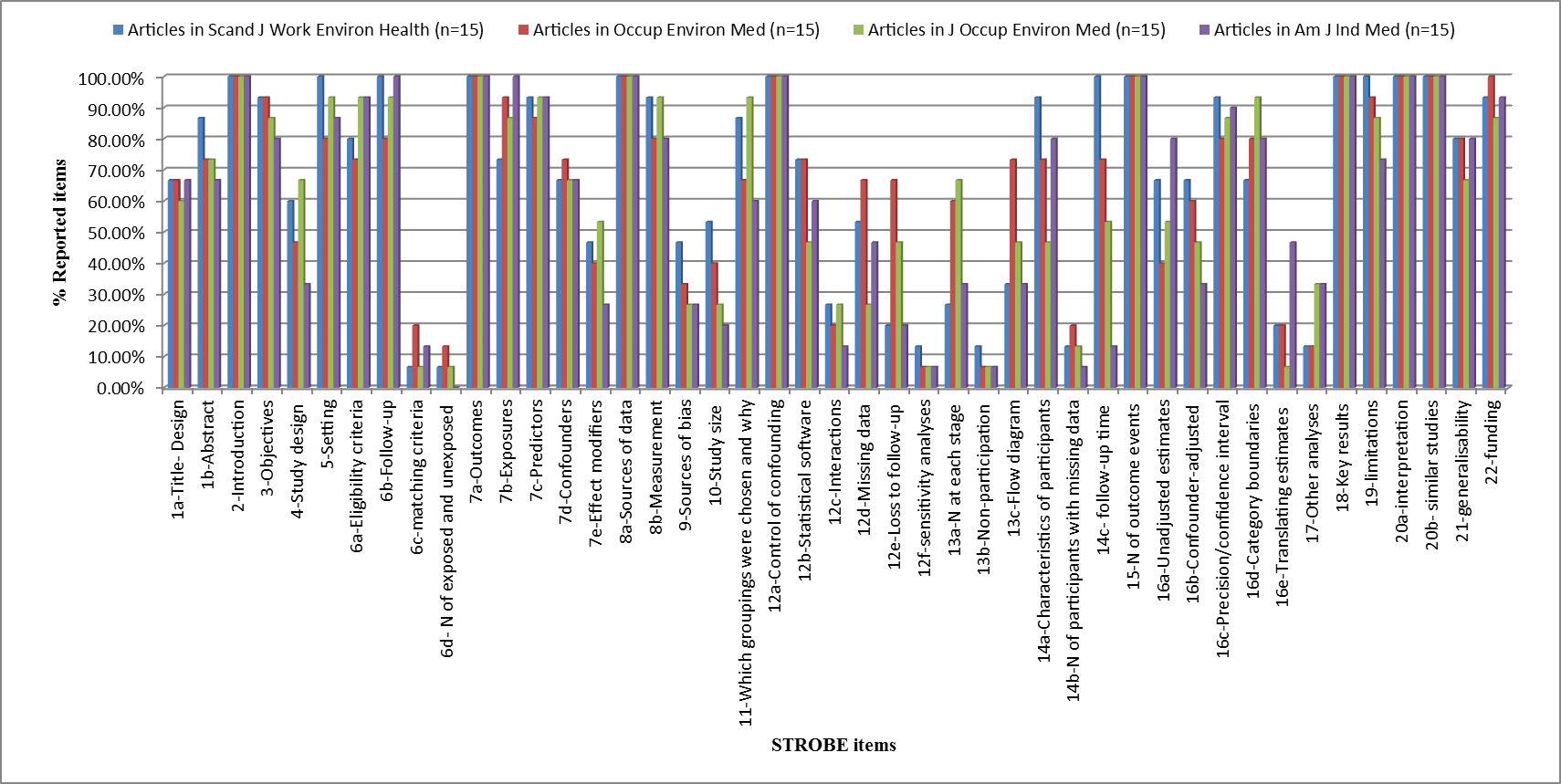

Supplement: Supplementary file 3 — Additional file 3: Figure S1. Percentages of adequately reported STROBE sub-items in articles published in the top four occupational medicine and health journals. [file 13104_2018_3367_MOESM3_ESM.docx]

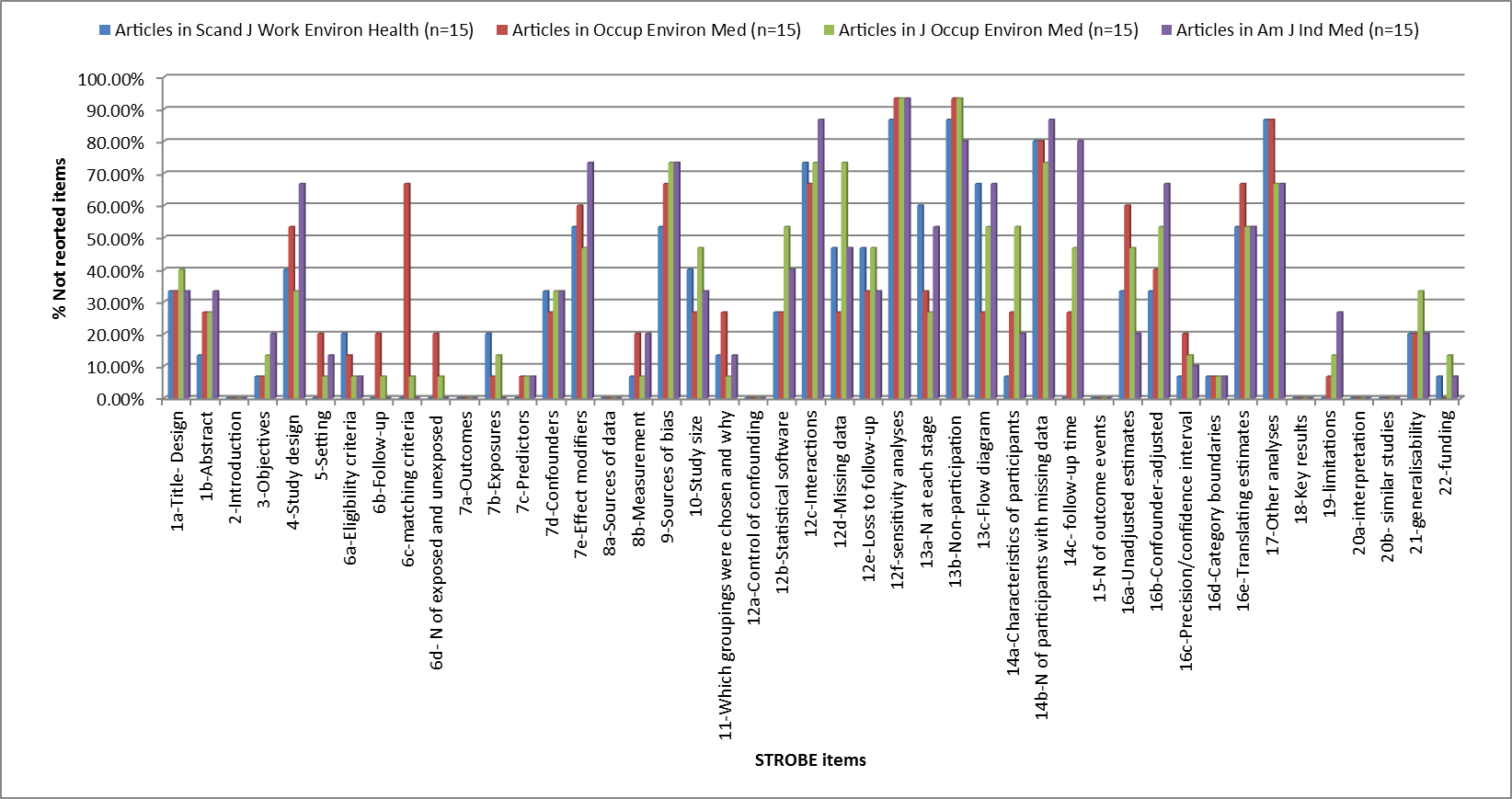

Supplement: Supplementary file 4 — Additional file 4: Figure S2. Percentages of STROBE items not adequately reported in articles published in the top four occupational medicine and health journals. [file 13104_2018_3367_MOESM4_ESM.docx]

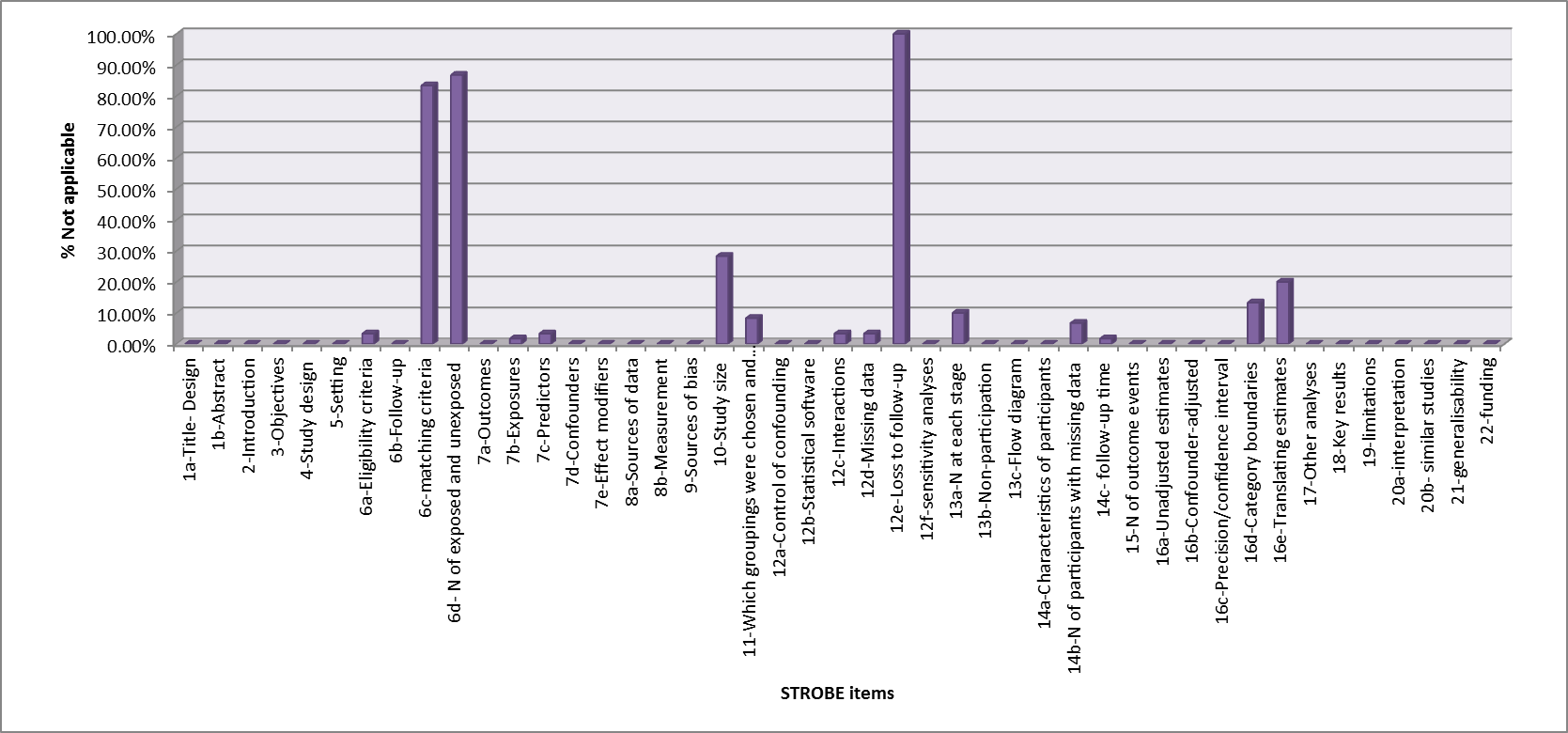

Supplement: Supplementary file 5 — Additional file 5: Figure S5. Percentages of not applicable STROBE items in articles published in the top four occupational medicine and health journals. [file 13104_2018_3367_MOESM5_ESM.docx]
